# White Blood Cells, COVID-19, and Mendelian Randomization

**DOI:** 10.3390/jpm12091425

**Published:** 2022-08-31

**Authors:** Cristian Baicus

**Affiliations:** 1 Faculty of Medicine, Carol Davila University of Medicine and Pharmacy, 050474 Bucharest, Romania; cristian.baicus@umfcd.ro; 2 Department of Internal Medicine, Colentina Clinical Hospital, 020125 Bucharest, Romania; 3 Clinical Research Unit, Réseau d’Épidémiologie Clinique International Francophone, 020125 Bucharest, Romania

**Keywords:** COVID-19, white blood cells, Mendelian randomization

## Abstract

Many observational studies have shown an association between the severity of COVID-19 and the different white blood cell counts, most frequently neutrophils, lymphocytes, and eosinophils. The studies aimed to predict the prognosis, and therefore, a causal relationship was unnecessary. However, if we begin to look at these biomarkers as potential therapeutic targets, then causality is essential. Observational studies cannot prove a causal relationship, and randomized trials are not always feasible. In this case, Mendelian randomization studies, considered more valid than observational studies, could add to the arguments for causality. Two Mendelian randomization studies tested for a causal relationship between the number of different white cell populations and COVID-19 severity, but their results are different; therefore, the problem of causality is not settled in this case.

COVID-19 was, at least until the appearance of Omicron variants, a disease with high mortality among patients with severe forms (respiratory failure). Many studies (most of them retrospective) tried to find the prognostic factors, either clinical (age and comorbidities) or in the laboratory. Studies found that the number of white blood cells was significantly associated with the severity of COVID-19 disease. Most studies that could link the white blood cells with a bad prognosis found that the patients with an elevated white blood cell count (most of them neutrophils) or a low count of lymphocytes were at a higher risk for a severe disease/death; therefore, the neutrophil-to-lymphocyte ratio appeared as an even better prognostic biomarker [1,2,3].

It was known before that eosinopenia was a marker of infection [4], and eosinopenia was associated with severe COVID-19 disease in most studies [5,6]. Most hospitalized COVID-19 patients have been found to have a degree of eosinopenia at admission irrespective of severity, and, in the patients with a good prognosis, the eosinophil count began to recover during the first week, while in the patients who died, eosinopenia was persistent to the end [7,8,9,10,11,12]. Some studies have even mentioned absolute/extreme eosinopenia if the eosinophil count was 0 [13,14]. Persistent eosinopenia and lymphopenia were associated with the cytokine storm, which appeared in patients with pulmonary involvement and severe disease [15]. Only a few studies on small samples showed that the severity of the disease was also associated with a decreased count of basophils [16].

In addition to these prognostic studies, at the beginning of the pandemic, diagnostic studies were also performed to more rapidly distinguish the patients infected with SARS-CoV-2 from those infected with other respiratory pathogens. Among the diagnostic tests, the hematological parameters were assessed, and the predictors for COVID-19 disease were generally the same as for severe COVID-19: lymphopenia, eosinopenia, and, rarely, basopenia [17,18].

Although at least some white blood cells were presumably involved in the pathogenesis of (severe) COVID-19, while other changes were only a result of inflammation, the authors of the clinical studies were not specifically concerned with a causal relationship: in diagnostic or prognostic studies, we are interested in predicting, regardless of the existence or not of confounding.

However, this changed when Sun et al. decided to evaluate causal associations and performed a Mendelian randomization study [19].

Typically, the more an association meets the Bradford Hill criteria [20], the more the association is causal. Among these criteria, one of the most important is the study design. The randomized trial provides the best evidence of causality, as it has the least bias. Unfortunately, this experimental approach is not always feasible, especially when regarding white blood cells’ characteristics and count as a cause. Therefore, all the cited studies above were observational, and most of them were retrospective.

Concerning validity, Mendelian randomization studies are located just under the randomized clinical studies and above the cohort studies, in terms of the hierarchy of studies, so they are considered more valid than the observational studies [21]. Although named “randomized”, they are not interventional/experimental studies, as there is no real randomization made by the investigators. The principle of these studies is that all traits are at least partially influenced by genetic effects and that, at conception, the genes from our parents are randomly transmitted to us (there is random segregation of alleles). Moreover, the genes influencing one trait are transmitted independently by the genes controlling any possible confounder. Therefore, the interference from other variables is equal between the groups, as in randomized clinical studies. In addition, in these studies, the temporality criterium of causality will always be respected, as the genes are fixed at conception.

However, the validity of Mendelian randomization studies depends on three main assumptions. The first one is that there is a strong association of the genetic variant with the risk factor of interest (relevance assumption) (for example, the low number of basophils from the study by Sun et al. [19]). This information usually comes from extensive genome-wide association studies (GWAS), searching for associations between genetic variants and numerous traits, and whose results are available in open databases.

The second assumption is that there are no unknown/unmeasured confounders of the associations between the genetic variant and outcome (independence assumption) and that the genetic variant is not associated with any of the confounding variables. Otherwise, confounding would not be eliminated, and the randomization would not have reached its aim. In our example, we should be sure that the genetic variants associated with the low basophil count leading to severe COVID-19 are not also associated with other risk factors for severe COVID-19. This assumption is never guaranteed, as we never know all the risk factors; therefore, it is impossible to verify the lack of association between an unknown confounder and the studied genetic variants, as is the case in observational studies, where it is possible to adjust only for the known/measured confounders.

The third assumption is that the genetic variants modify the outcome only by the risk factor of interest (in our example, the genetic variants associated with basopenia do not contribute to severe COVID-19 by an alternative pathway, other than basopenia)—in other words, there is no pleiotropy.

While the first assumption can be tested in the GWAS, the other two assumptions cannot be checked with certainty, although sophisticated methodology and statistics exist for this purpose.

A systematic review searching for studies until December 2021 found 50 studies using Mendelian randomization in the quest for causal relationships between different risk or prognostic factors for COVID-19 [22]. Two of the studies looked for hematologic parameters as biomarkers for COVID-19 incidence and severity, and the conclusion was that “there is suggestive evidence showed that hematological traits (higher basophil count, basophil percentage of white cells, lymphocyte count, myeloid white cell count, neutrophil count…) were associated with reduced risk of COVID-19 severity and hospitalization” [22].

However, reading the two studies, one can see that Sun et al. found basophil and myeloid counts associated with COVID-19 prognosis (and did not find any association between disease severity and total white blood cell, neutrophil, eosinophil, or lymphocyte counts) [19]. In contrast, Wang et al. found that white blood cell, neutrophil, and lymphocyte counts were associated with COVID-19 severity, but not eosinophil, basophil, or monocyte count [23]. This discrepancy shows that Mendelian randomization is not a foolproof method, and the results of the studies must be judged in context.
